# Hydroisomerization of n-Hexadecane Over Nickel-Modified SAPO-11 Molecular Sieve-Supported NiWS Catalysts: Effects of Modification Methods

**DOI:** 10.3389/fchem.2022.857473

**Published:** 2022-04-07

**Authors:** Xiaojun Dai, Yan Cheng, Meng Si, Qiang Wei, Yasong Zhou

**Affiliations:** State Key Laboratory of Heavy Oil Processing, China University of Petroleum, Beijing, China

**Keywords:** Ni-modified, SAPO-11, NiWS-supported catalyst, active phase, hydroisomerization

## Abstract

The complexation-excessive impregnation modification method, which was original in this study, and the ion-exchange method and the *in situ* modification method were used to synthesize Ni-modified SAPO-11 molecular sieves. With the Ni-modified SAPO-11 samples as support, the corresponding NiWS-supported catalysts for the hydroisomerization of n-hexadecane were prepared. The effects of Ni-modification on SAPO-11 characteristics and the active phase were studied. The structure, morphology, and acidity of SAPO-11, as well as the interaction between active metals and support, the morphology, dispersibility, and stacking number of the active phase, were all changed by Ni-modification methods. The complexation-excessive impregnation modification method deleted a portion of Al from SAPO-11 molecular sieves while simultaneously integrating Ni into the skeletal structure of the surface layer of SAPO-11 molecular sieves, considerably enhancing the acidity of SAPO-11 molecular sieves. Furthermore, during dealumination, ethylenediaminetetraacetic acid generated more mesoporous structures and increased the mesoporous volume of SAPO-11 molecular sieves. Because the complexation-excessive impregnation modification method increased the amount of Ni in the surface framework of the SAPO-11 molecular sieve, it has weakened the interaction between the active phase and the support, improved the properties of the active phase, and greatly improved the hydroisomerization performance of NiW/NiSAPO-11. The yield of i-hexadecane of NiW/NiSAPO-11 increased by 39.3% when compared to NiW/NiSAPO-11. It presented a realistic approach for increasing the acidity of SAPO-11, reducing the interaction between active metals and support, and improving the active phase stacking problem.

## Introduction

The presence of long-chain n-alkanes is thought to be the cause of the high freezing point of diesel and the poor low-temperature fluidity of lubricating base oil (Tao et al., 2017). The formation of branched isomers by hydroisomerization of long-chain n-alkanes can effectively lower the freezing point of diesel and increase the low-temperature fluidity of lubricating base oil (Du et al., 2018). The bifunctional catalyst for the hydroisomerization of n-alkanes contains an acid site for the skeletal isomerization of olefin intermediates and a metal site for de/hydrogenation (Wei et al., 2017). Acidic supports, such as zeolites, usually supply acid sites, whereas noble metals or other transition metals give metal sites (Yang et al., 2017a). Because of their one-dimensional porosity and low acidity, SAPO-11 molecular sieves are commonly employed as a support for n-alkane hydroisomerization catalysts (Guo et al., 2012). Noble metals like platinum and palladium have strong de/hydrogenation activities, whereas nonnoble metals like nickel are also used as metal components in hydroisomerization catalysts (Zhao et al., 2014; Wang et al., 2018).

Although SAPO-11 molecular sieves have been used in industry and have shown to be a solid support for hydroisomerization catalysts, increasing their performance in hydroisomerization is still a concern worth investigating (Blasco et al., 2006). The active sites for catalyzing isomerization of the alkane molecular skeleton are thought to be Brnsted (B) acid sites in the SAPO-11 molecular sieve, particularly the medium strength B acid sites (Guo et al., 2013). As a result, improving the isomerization performance of SAPO-11 requires a better understanding of enhancing the proportion of medium and strong B acid sites. Lyu et al. (2019a) used *in situ* synthesis to introduce varying amounts of Ni into SAPO-11 to investigate the effects of metal-acid balance on n-hexane hydroisomerization and the acidity of SAPO-11. However, up to now, no one has studied the effects of modification methods in the process of Ni modification on the physicochemical properties (such as textural and acidity) of SAPO-11 molecular sieves. Furthermore, despite the remarkable catalytic activity of noble metals as metal components of hydroisomerization catalysts, there are two issues. On the one hand, the high cost of noble metals restricts their use in industry (Tian and Chen., 2014). Noble metals, on the other hand, are poisoned and inactivated due to their sensitivity to sulfur-containing compounds in raw materials (Yang et al., 2017b; Lyu et al., 2020). Although some researchers have reported employing Ni as a metal component instead of noble metals, hydroisomerization results revealed that Ni has a high hydrogenolysis activity, resulting in lower isomer selectivity and yield than noble metal-supported catalysts (Karthikeyan et al., 2016; Yang et al., 2017c). Transition metal sulfides have a good de/hydroisomerization performance and are widely used as the active phase of catalysts for hydrodesulfurization (Díaz de León et al., 2017), hydrodenitrogenation, and hydrocracking (Cui et al., 2019), such as NiMoS (Zhou et al., 2017) and NiWS (Minaev et al., 2019). In the past, there have been many reports on the application of noble metal (Pt, Pd) or Ni-supported catalysts in alkane hydroisomerization (Lyu et al., 2017; Hongloi et al., 2021), but there have been few on the application of transition metal sulfide catalysts in alkane hydroisomerization and the effects of the properties of the transition metal sulfide active phase on alkane hydroisomerization (Shi et al., 2009; Tan et al., 2021).

In this study, the ion-exchange method, the *in situ* modification method, and the complexation-excessive impregnation modification method were used to modify SAPO-11 molecular sieves with Ni in this study. The hydroisomerization of n-hexadecane was carried out using the Ni-modified SAPO-11 molecular sieves, which were supported by NiWS. The impact of various modification methods on the physicochemical properties of SAPO-11, the active phase properties, and the hydroisomerization performance of several catalysts were studied.

## Experimental

### Materials

Phosphoric acid (H_3_PO_4_, 85wt%; Aladdin), pseudo-boehmite (Al_2_O_3_, 70wt%; Macklin), acid silica sol (SiO_2_, 30wt%; Dezhou Jinghuo technology Glass Co., Ltd.), di-n-propylamine (DPA, 99wt%; Aladdin), diisopropylamine (DIPA, 99wt%; Aladdin), dodecyltrimethylammonium bromide (DTAB, 99wt%; Aladdin), ammonium chloride (NH_4_Cl, 99.5wt%; Macklin), nickel nitrate hexahydrate(Ni(NO_3_)_2_·6H_2_O, 98wt%; Aladdin), ammonium metatungstate hydrate ((NH_4_)_6_H_2_W_12_O_40_·xH_2_O, 99.5wt%; Macklin), ethylenediaminetetraacetic acid (EDTA, 98wt%; Aladdin), and deionized water.

### Synthesis and Modification of SAPO-11 Molecular Sieves

Deionized water and phosphoric acid were mixed in a normal synthesis procedure, then pseudo-boehmite was added to the solution and agitated for 2 h. DPA and DIPA were added and mixed continuously for 2 h. Drop by drop, acid silica sol was added to the system and aggressively agitated for 2 h. Finally, DTAB was added and agitated for 1 h, resulting in an initial gel with the following molar composition: 1.0 Al_2_O_3_: 0.75 P_2_O_5_: 0.45 SiO_2_: 0.5 DPA: 0.5 DIPA: 0.05 DTAB: 45 H_2_O. The gel was pre-crystallized at 90°C for 12 h before being crystallized at 190°C for 24 h. The SAPO-11 molecular sieves were obtained by washing the solid products collected by filtration to neutrality with deionized water, drying them at 110°C overnight, and calcining them at 600°C for 6 h.

For the ion-exchange procedure, 10.0 g of SAPO-11 was mixed with 100 g of 0.3 mol/L nickel nitrate solution, which was then exchanged at 90°C for 4 h, filtered, dried at 110°C overnight, and calcined at 500°C for 4 h. Ni@SAPO-11 was the name given to the dried and calcined sample. The operation procedures for the *in situ* modification method were similar to the above-mentioned SAPO-11 synthesis steps, with the exception that 3% nickel was added during the initial gel formation process (the molar ratio of Ni/Al_2_O_3_ was 3%). The products were filtered before being dried at 110°C overnight and calcined for 4 h at 500°C. Ni-SAPO-11 was the name given to the dried and calcined sample. 0.1 mol EDTA was added to 50 ml of 0.3 mol/L nickel nitrate solution for the complexation-excessive impregnation modification procedure, and then 10 g of SAPO-11 molecular sieve particles with 20–40 mesh were impregnated with the resulting solution. SAPO-11 molecular sieve particles after complexation-excessive impregnation were obtained by filtration, dried, and calcined, and designated NiSAPO-11 after soaking for 4 h.

### Preparation of NiW-Supported Catalysts

The SAPO-11, Ni@SAPO-11, and Ni-SAPO-11 particles were pressed, crushed, and sieved to a size of 20–40 mesh. The NiW-supported catalysts were made using the incipient-wetness impregnation method with an aqueous solution of nickel nitrate hexahydrate and ammonium metatungstate hydrate, then dried at 110°C for 6 h and calcined at 500°C for 4 h after being evaporated at room temperature overnight. The loading concentration of NiO was 5% and the loading concentration of WO_3_ was 15% for each catalyst. NiW/SAPO-11, NiW/Ni@SAPO-11, NiW/Ni-SAPO-11, and NiW/NiSAPO-11 were the names given to the resulting catalysts.

### Characterization

The SAPO-11 samples were characterized by X-ray diffraction (XRD) on a Bruker AXS D8 Advance X-ray diffractometer using Cu Kα radiation at 40 kV and 40 mA, and 2*θ* varied from 5 to 90° at a scanning speed of 5°/min. On a field-emission environmental scanning electron microscope, images of the SAPO-11 samples were taken using scanning electron microscopy (SEM) (FEI Quanta 200F). After degassing the materials at 350°C under vacuum for 15 h, N_2_ adsorption-desorption measurements were performed on a Micromeritics ASAP 2020 analyzer at −196°C. The Brunauer-Emmett-Teller (BET) and de Boer *t*-plot methods were used to compute the specific surface area and micropore volume, respectively, while the Barrett-Joyner-Halenda technique was used to calculate the mesopore volume. A Micromeritics auto-chem 2920 device was used to analyze temperature-programmed desorption of ammonia (NH_3_-TPD). The sample was heated to 600°C in an Ar flow for 30 min, then switched to an ammonia flow for another 30 min before being cooled to 70°C. The sample was purged with Ar for 2 h to eliminate the physically adsorbed ammonia, and the TPD signal was recorded using a thermal conductivity detector with a heating rate of 10°C/min from 70 to 600°C. A Nicolet 5700 spectrometer was used to record Pyridine adsorbed infrared (Py-IR) spectra. At 200 and 350°C, the pyridine samples were evacuated. On a Quantachrome Autosorb-iQ-C chemical adsorption system, temperature-programmed hydrogen reduction (H_2_-TPR) measurements were taken. The samples were heated at a rate of 10°C/min in an H_2_-Ar flow containing 5% H_2_ from room temperature to 1,050°C. The presulfurized catalysts were analyzed using X-ray photoelectron spectroscopy (XPS) on a Thermo spectrometer using Al Kα radiation as the excitation light source. To calibrate the binding energy scale, all spectra used the Al 2p peak with a binding energy of 74.6 eV. The presulfurized catalysts’ XPS spectra were decomposed using XPS PEAK, and the deconvolution was achieved using Gaussian-Lorentzian band shapes. A JEM 2100 LaB_6_ transmission electron microscope was used to obtain high-resolution transmission electron microscope (HRTEM) images of the presulfurized catalysts. The average length and average stacking number of WS_2_ slabs were determined using methods described in the literature (Yu et al., 2012):

$$\text{Average slab length}\;\bar{L}=\frac{\sum_{i=1}^{n}n_{i}l_{i}}{\sum_{i=1}^{n}n_{i}}$$ (1)

$$\text{Average stacking number }\bar{N}=\frac{\sum_{i=1}^{n}n_{i}N_{i}}{\sum_{i=1}^{n}n_{i}}$$ (2)

where *l*_*i*_ denotes the WS_2_ slab length, *n*_*i*_ denotes the number of slabs of length *l*_*i*_, and *N*_*i*_ is the number of layers in a WS_2_ slab. The dispersion degree of the WS_2_ active phase, *f*_*W*_, was derived using the following equation, assuming that the WS_2_ slabs are presented as perfect hexagons (Tao et al., 2014):

$$f_{W}=\frac{W_{edge}}{W_{total}}=\frac{\sum_{i=1}^{t}6\left(n_{i}-1\right)}{\sum_{i=1}^{t}\left(3n_{i}^{2}-3n_{i}+1\right)}$$ (3)

where *W*_*edge*_ denotes the W atoms on the edges of WS_2_ slabs, *W*_*total*_ denotes the total number of W atoms, *n*_*i*_ denotes the number of W atoms along one side of a WS_2_ slab determined by its length [L = 3.2 (2ni—1) Å], and *t* denotes the total number of slabs determined by at least 500 WS_2_ slabs obtained from HRTEM images of various catalysts.

### Catalytic Performance Assessment

In a fixed-bed hydrogenation micro-reactor, the hydroisomerization of n-hexadecane (n-C_16_) was carried out. In a typical reaction, the tubular furnace held 9.0 ml of catalyst and 22 ml of silica sand. The catalyst was presulfurized using a CS_2_ cyclohexane solution containing 2% CS_2_ at 320°C and 4 MPa for 5 h with a liquid hourly space velocity (LHSV) of 7 h^−1^ and an H_2_/oil ratio of 100 (v/v) before the reaction. After presulfurization, the catalytic performance of the catalyst was examined at 2 MPa, 1.5 h^−1^ LHSV, 600 (v/v) H_2_/oil, and 320–400°C reaction temperature. When the temperature was reduced to the reaction temperature, a syringe pump was used to feed the reactant n-hexadecane into the reactor. The products were analyzed using a Shimadzu GC-2014 gas chromatograph with a capillary HP-PONA column and GC-MS for qualitative analysis. The TOF (turnover frequency, which is used to evaluate the catalytic activity of a catalyst) of each active site was calculated using the following equation based on the number of all available active sites and the conversion of n-C_16_ of each catalyst at 340°C (Wen et al., 2020):

$$TOF=\frac{V_{feed}\cdot x}{n_{W}\cdot f_{W}}$$ (4)

where *V*_*feed*_ is the feed rate of the reactant n-C_16_ in mol/h, *x* is the conversion of n-C_16_ at 340°C, *n*_*w*_ is the amount of W atom in the catalyst in mol, and *f*_*W*_ is the dispersion degree of W species. The following equation was used to compute the hydroisomerization reaction rate constant (Pimerzin et al., 2019):

$$k_{iso}=\frac{V_{feed}}{m}\ln\left(1-x\right)$$ (5)

where *k*_*iso*_, *V*_*feed*_, *m* and *x* are the quasi-first-order reaction rate constant of n-C_16_ hydroisomerization on different catalysts, the feed flow rate of the reactant n-C_16_ in mol/h, the mass of catalyst and the conversion of n-C_16_, respectively.

## Results and Discussion

### Phase Structure

**FIGURE 1:**
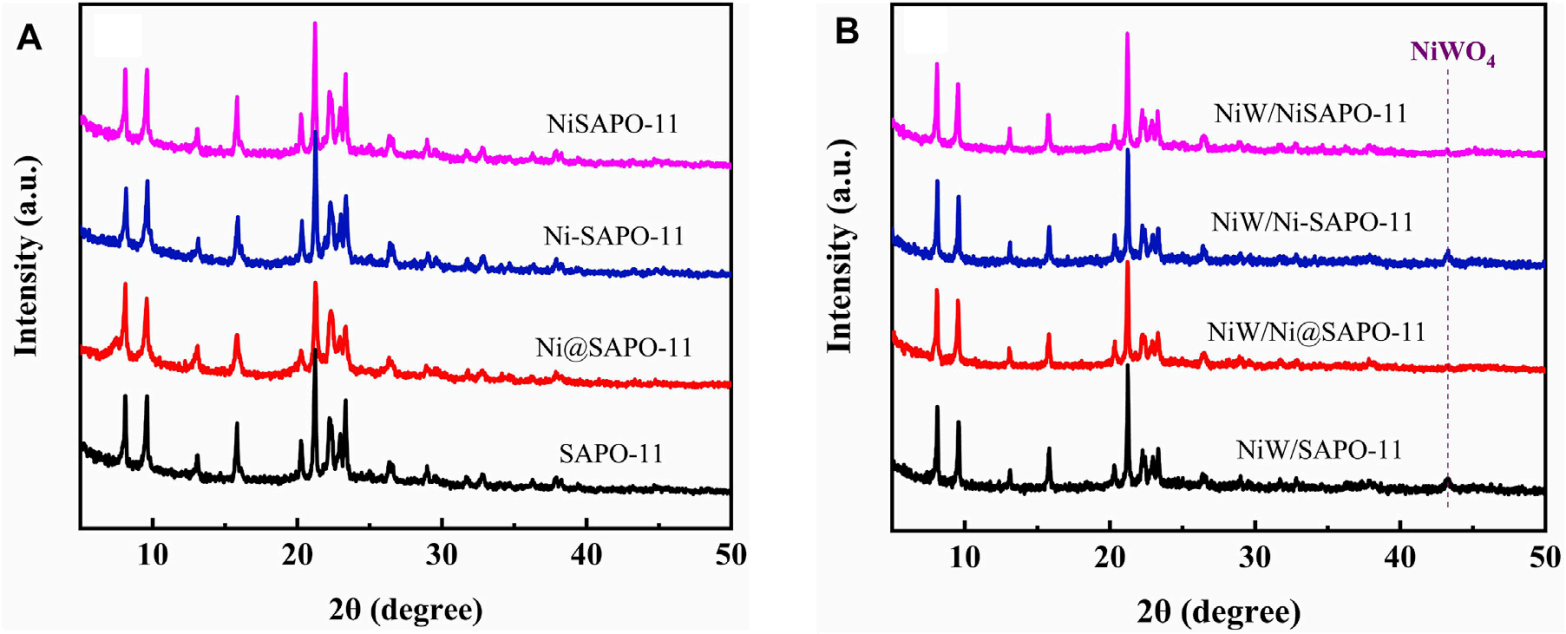
XRD patterns of different SAPO-11 molecular sieves **(A)** and different catalysts **(B)**.

The XRD patterns of Ni-modified SAPO-11 molecular sieves generated by various ways are shown in Figure 1A. All samples exhibited diffraction peaks in the 2θ range of 5–50° that were assigned to the normal AEL structure, demonstrating that SAPO-11 samples retained full structural units after Ni alteration by various methods (Lyu et al., 2019b). The prominent characteristic diffraction peaks of Ni-SAPO-11 indicate that it has a high crystallinity (Song et al., 2017). Because Ni species replaced a portion of Al species in the SAPO-11 framework, crystalline Ni species resulted. The strength of the distinctive diffraction peaks of Ni@SAPO-11 reduced slightly when compared to SAPO-11, which could be attributable to a decrease in crystallinity during the ammonium-exchange and nickel ion-exchange processes (rehydration) (Yang et al., 2017c). Furthermore, the intensity of the characteristic diffraction peaks of NiSAPO-11 did not significantly decrease, indicating that EDTA could remove some Al species but not the crystal structure of SAPO-11. A weak characteristic diffraction peak attributed to NiO appeared at 2θ = 37.2° of XRD patterns of all the samples, which indicates that there were a small number of NiO species in all the samples, and the distribution of NiO species in SAPO-11 molecular sieves was relatively uniform. Figure 1B shows the XRD spectra of the different catalysts. It can be seen that the different catalysts still retained the characteristic diffraction peaks attributed to AEL structure, indicating that the loading of metal components did not cause the crystal structure of SAPO-11 molecular sieves to be destroyed. In addition, all the catalysts showed a weak characteristic diffraction peak attributed to NiWO_4_ species around 2θ = 43.2°, which indicates that NiWO_4_ species existed in all samples and dispersed relatively evenly.

### Morphology

**FIGURE 2:**
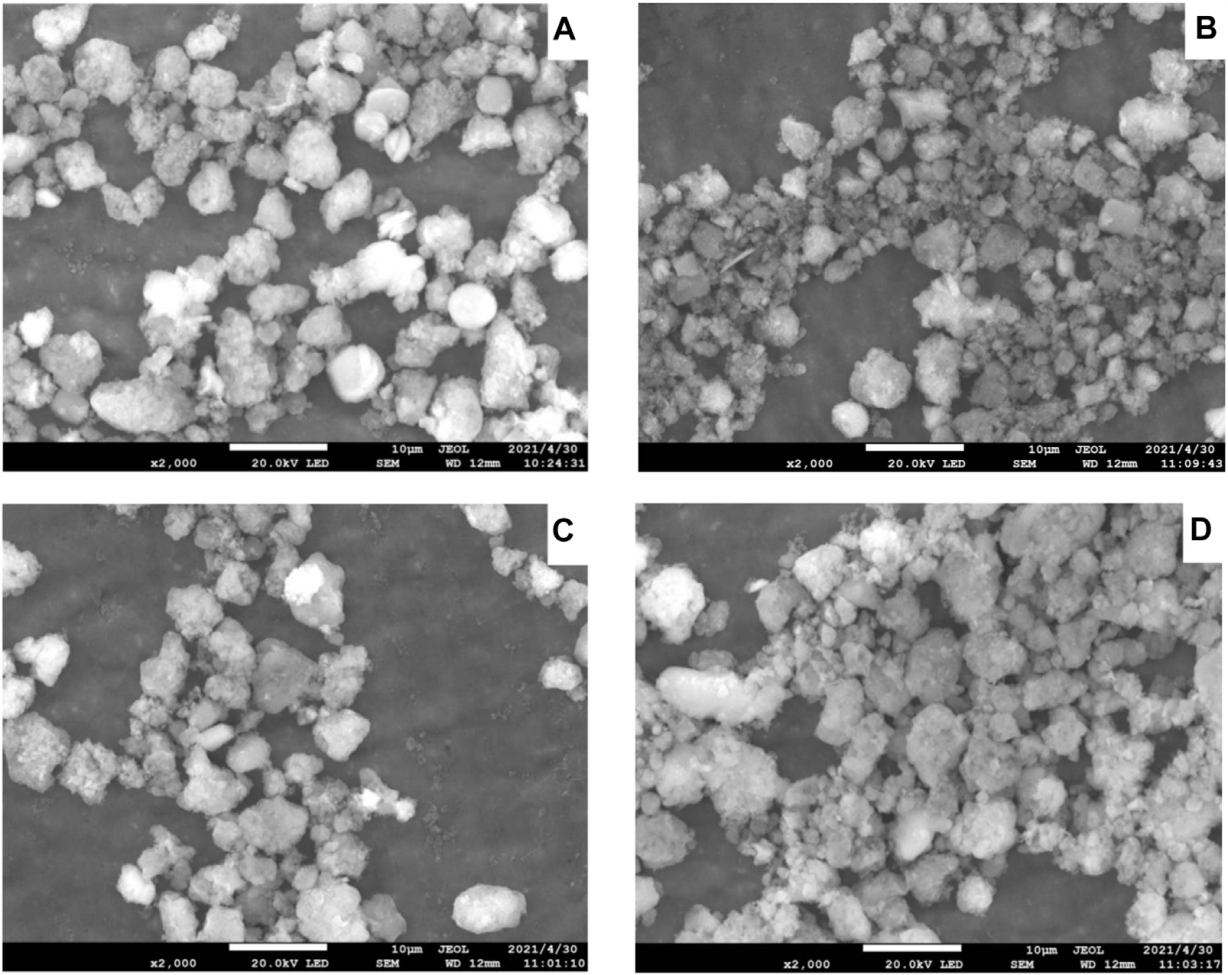
SEM images of Ni-modified SAPO-11: **(A)** SAPO-11, **(B)** Ni@SAPO-11, **(C)** Ni-SAPO-11, and **(D)** NiSAPO-11.

SEM images of Ni-modified SAPO-11 molecular sieves generated by various procedures are shown in Figure 2. SAPO-11 had pseudospherical particles with a particle size of roughly 5 μm and a fairly uniform particle size distribution. The particle shape of Ni@SAPO-11 was not as regular as that of SAPO-11, and the discrepancy could be due to its particle form being destroyed when it was exchanged in aqueous solution twice. While Ni-SAPO-11 was basically the same as SAPO-11 in particle shape and size. As for NiSAPO-11, a small number of small particles similar to amorphous substances appear in its SEM image, which may be the smaller particles formed by the decomposition of SAPO-11 molecular sieve particles in the process of removing Al species by EDTA.

### Textural Properties

**FIGURE 3:**
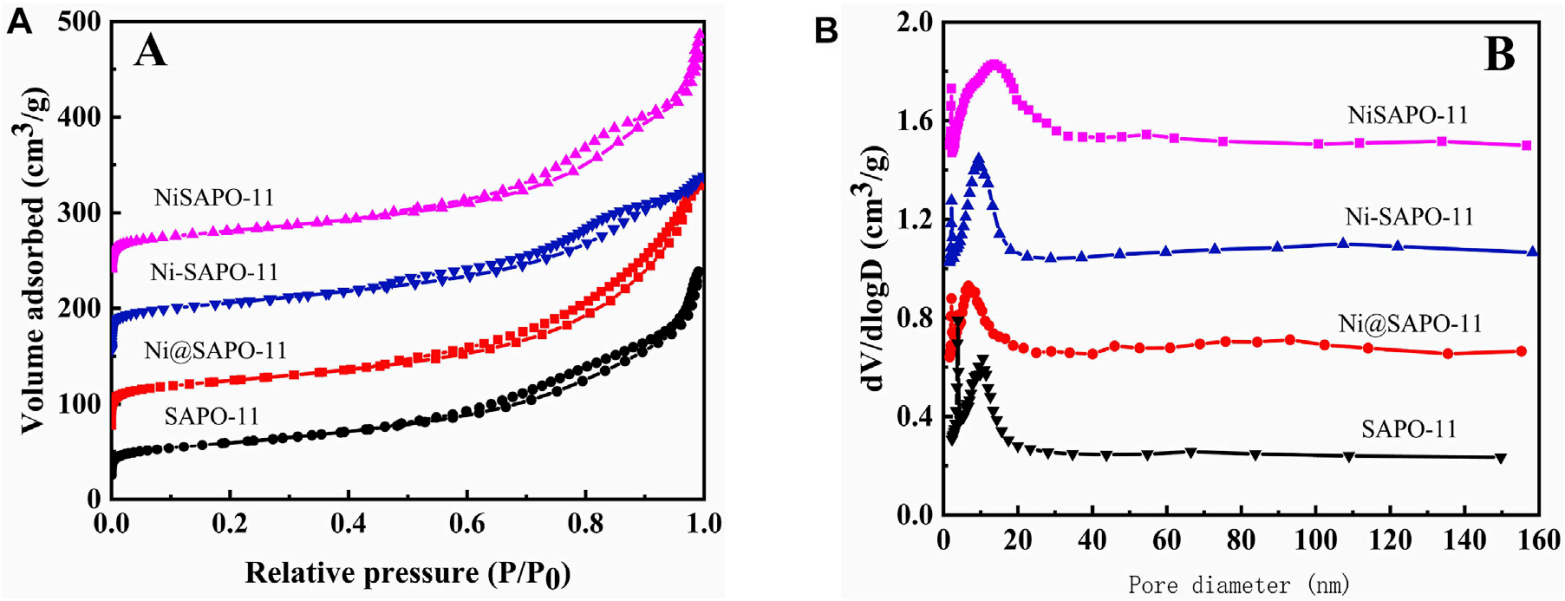
N_2_ adsorption-desorption isotherms **(A)** and pore size distribution **(B)** of Ni-modified SAPO-11 molecular sieves.

Figure 3 shows the N_2_ adsorption-desorption isotherms (Figure 3A) and pore size distribution (Figure 3B) of the Ni-modified SAPO-11 molecular sieves. Isotherms with typical H4-type hysteresis loops were seen in all samples (Chen et al., 2017). At low relative pressure (10^–5^ ≤ P/P_0_ ≤ 10^–2^), the amount of N_2_ adsorption in all samples rose dramatically, which was attributable to N_2_ filling the micropores (Du et al., 2019). Within the relative pressure P/P_0_ range of 0.4–0.9, all samples showed clear hysteresis loops, showing that these samples have a lot of mesopores (Wen et al., 2019). The hysteresis loop of NiSAPO-11 is clearly larger than that of other samples, indicating that NiSAPO-11 has a more mesoporous structure than other samples. This is because, when eliminating Al species, EDTA dissolved and etched in the crystals of SAPO-11 molecular sieves, forming many new mesoporous structures. Figure 3B shows the pore size distribution of all samples, with the pore size of SAPO-11, Ni@SAPO-11, and Ni-SAPO-11 primarily dispersed around 12 nm. The foregoing findings show that these samples include hierarchical micro-mesoporous structures. However, the mesoporous size of NiSAPO-11 is centered at 18 nm, which is larger than that of other samples, implying that EDTA does have a pore-expansion role.

The BET surface area (S_BET_), exterior surface area (S_ext_), and pore volume of the Ni-modified SAPO-11 are shown in Table 1. The S_BET_ and pore volume of Ni@SAPO-11 were reduced to some extent when compared to SAPO-11. One probable explanation is that Ni species enter the pores, blocking certain micropores and occupying a portion of the mesopore volume (*V*_meso_) in the ion-exchange process. Another possibility is that during the ion-exchange process, a portion of the crystal structure collapses, causing the pore channels to fill. The S_BET_ and V_meso_ of Ni-SAPO-11 have increased, indicating that the addition of Ni *in situ* helps to improve the *S*_BET_ and *V*_meso_ of SAPO-11 (Liu et al., 2013). The micropore volume (V_micro_) of Ni-SAPO-11 decreased, implying that some Ni species remain in the micropores and occupy the micropore volume. The S_BET_, V_micro_, and V_meso_ of NiSAPO-11 all increased, with V_meso_ increasing by a substantial margin, confirming the prior conclusion that EDTA has the ability to ream and manufacture new mesopores. The S_BET_ and V_meso_ of the different SAPO-11 samples increased in the order of Ni@SAPO-11 < SAPO-11 < Ni-SAPO-11 < NiSAPO-11, indicating that the textural properties of SAPO-11 molecular sieves can be improved most obviously by the complexation-excessive impregnation modification method.

**TABLE 1:** Textural properties of Ni-modified SAPO-11.

Sample	S_BET_, m^2^/g	S_ext_, m^2^/g	V_micro_, cm^3^/g	V_meso_, cm^3^/g	V_total_, cm^3^/g
NiSAPO-11	188	96	0.11	0.36	0.47
Ni-SAPO-11	174	94	0.08	0.30	0.38
Ni@SAPO-11	158	88	0.07	0.20	0.27
SAPO-11	166	91	0.09	0.26	0.35

### Acidity Properties

**FIGURE 4:**
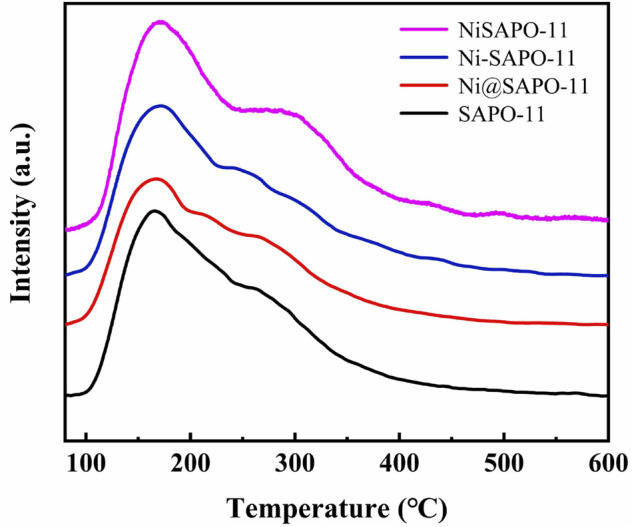
NH_3_-TPD profiles of Ni-modified SAPO-11 molecular sieves.

The NH_3_-TPD measurements were used to determine the acid amounts and acid strength distribution of the Ni-modified SAPO-11 molecular sieves manufactured using various procedures. Figure 4 shows the NH_3_-TPD profiles of the several Ni-modified SAPO-11 samples. Each NH_3_-TPD profile revealed two NH_3_ desorption peaks about 180 and 310°C, corresponding to NH_3_ adsorbed on weak acid sites, medium and strong acid sites, respectively (Yang et al., 2019). The intensity of the desorption peak represents the acid amount (Li and Wu, 2020). The acid amount of Ni@SAPO-11 was much lower than that of SAPO-11, which was attributable to the fact that part of the acid sites were covered and sheltered by Ni species during Ni ion exchange. The strength of the NH3 desorption peak corresponding to weak acid sites reduced somewhat in Ni-SAPO-11, indicating that the weak acid amount of Ni-SAPO-11 fell slightly (Yang et al., 2017c). This is due to the fact that the acid sites in SAPO-11 are thought to be formed by Si-OH, Al-OH, P-OH, and Si-OH-Al, respectively (Tiuliukova et al., 2018). Some Al precursors were substituted with Ni precursors, resulting in a reduction in acid sites. The intensities of the NH_3_ desorption peaks corresponding to medium and strong acid sites increased, indicating that the concentrations of medium and strong acid increased as well. It can be explained by the fact that Ni has a greater average electronegativity (1.91) than Al (1.71), and Ni has a higher covalency than Al. As a result, the Brnsted protons and Lewis caverns generated by Ni substituting part of Al in SAPO-11 have a higher acid density, resulting in more medium and strong acid sites in Ni-SAPO-11. The amount of weak acid, medium and strong acid in NiSAPO-11 were all improved. The increase in weak acid amonut is due to EDTA dredging some blocked channels and establishing new ones, exposing and detecting more acid sites to a large extent. The rise in medium and strong acid amounts in NiSAPO-11 can be attributed to two factors. On the one hand, the exposure of acid sites is caused by an increase in pore volume. In the process of eliminating Al species, EDTA can integrate Ni species into the surface framework of SAPO-11 molecular sieves to generate Ni-OH-Si species, which helps to enhance the density of medium and strong acid in SAPO-11. The results show that the amounts of the weak acid in Ni-modified SAPO-11 decreased in the order of NiSAPO-11 > SAPO-11 > Ni@SAPO-11 > Ni-SAPO-11, while the amounts of medium and strong acid decreased in the order of NiSAPO-11 > Ni-SAPO-11 > SAPO-11 > Ni@SAPO-11.

**FIGURE 5:**
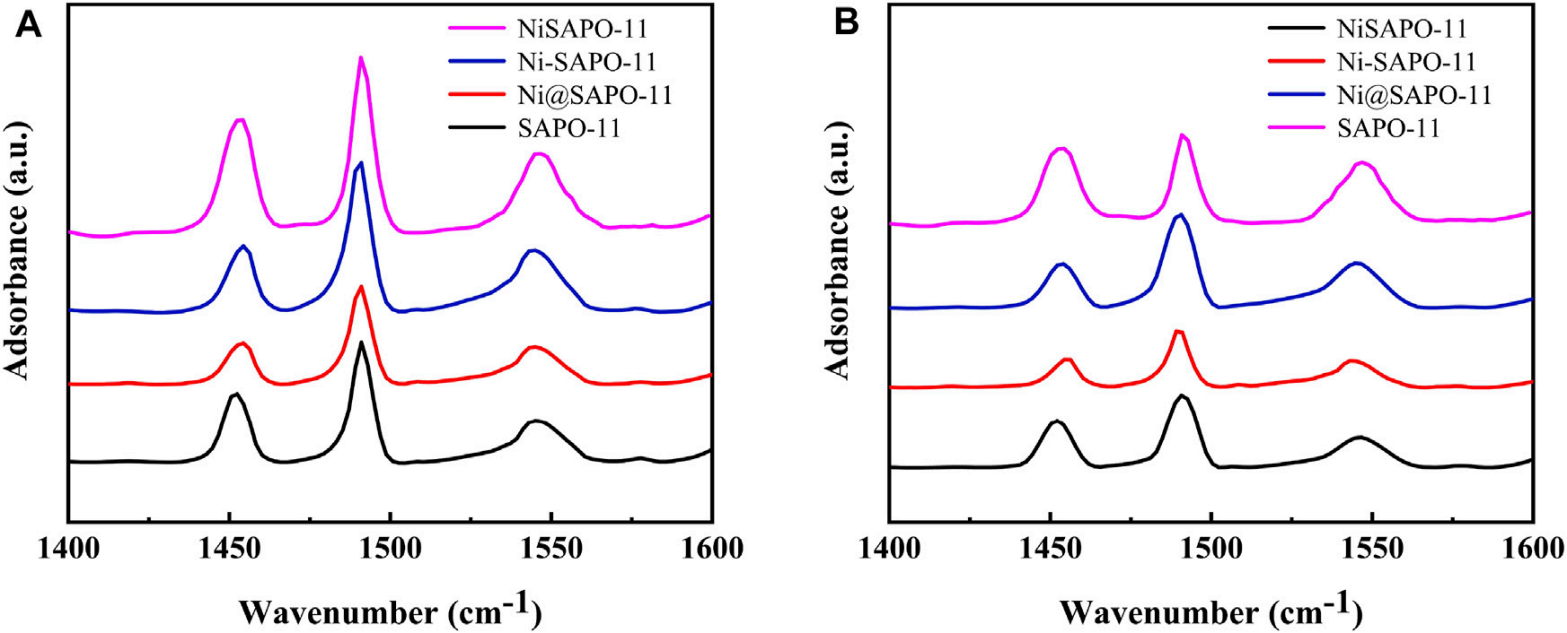
Py-IR spectra of Ni-modified SAPO-11 molecular sieves at 200°C **(A)** and 350°C **(B)**.

Py-IR was used to analyze the variations in Brønsted (B) acid sites and Lewis (L) acid sites in the Ni-modified SAPO-11 molecular sieves to determine the acidity of the different SAPO-11 samples (Jin et al., 2018). Figure 5 shows the Py-IR spectra of the Ni-modified SAPO-11 samples generated using various procedures. Py-IR spectra obtained after pyridine molecules desorbed at 200°C were used to compute the amount of weak acid, whereas Py-IR spectra formed after pyridine molecules desorbed at 350°C were used to calculate the amounts of medium and strong acid. Table 2 shows the results of calculating the number of B acid sites and L acid sites and the accompanying findings. All samples had three peaks in the range of 1,400–1,600 cm^−1^, as illustrated in Figure 5. The peaks at 1,455 cm^−1^ and 1,545 cm^−1^ are assigned to pyridine molecules desorbed on L acid sites and B acid sites, respectively (Wen et al., 2020), whereas the peak at 1,490 cm^−1^ is attributed to a combined line of B acid sites and L acid sites (Zhang et al., 2018). The amounts of weak L acid in Ni-modified SAPO-11 molecular sieves decreased in the following order: NiSAPO-11(56.88 μmol/g) > Ni-SAPO-11 (35.84 μmol/g) > SAPO-11 (29.32 μmol/g) > Ni@SAPO-11 (24.25 μmol/g), while the amounts of medium and strong L acid decreased in the following order: NiSAPO-11 (24.66 μmol/g) > SAPO-11 (20.97 μmol/g) > Ni-SAPO-11 (18.16 μmol/g) > Ni@SAPO-11 (15.11 μmol/g). This can be explained by the fact that during the ion-exchange and *in situ* modifications, certain NiO species covered several strong L acid sites. However, some additional weak L acid sites were generated in Ni species in the surface framework of NiSAPO-11, resulting in an increase in the amount of weak L acid and the amount of medium and strong Lacid in NiSAPO-11. The amounts of weak B acid in Ni-modified SAPO-11 molecular sieves decreased in the order of SAPO-11 (91.87 μmol/g) > NiSAPO-11 (82.21 μmol/g) > Ni-SAPO-11 (69.91 μmol/g) > Ni@SAPO-11 (53.09 μmol/g), while the amounts of medium and strong B acid decreased in the order of NiSAPO-11 (58.35 μmol/g) > Ni-SAPO-11 (48.21 μmol/g) > SAPO-11 (30.62 μmol/g) > Ni@SAPO-11 (23.51 μmol/g). In the process of Ni ion exchange (part of H^+^ being swapped by Ni^2+^), Ni species covered some weak B acid sites and medium and strong B acid sites, resulting in a decrease in both the amount of weak B acid and the amount of medium and strong B acid (MSB) in Ni@SAPO-11. Some Ni species would undoubtedly cover the acid sites during Ni *in situ* alteration, resulting in a reduction in the number of weak B acid sites in Ni-SAPO-11. Meanwhile, Ni species entered the skeleton structure of SAPO-11 and substituted Al species by mechanism (1) and mechanism (2). Whether it is (NiO_4/2_)^2−^(PO_4/2_)^+^ species produced by mechanism (1) substitution or (HONiO_4/2_)^2−^(PO_4/2_)^+^ species produced by mechanism (2) substitution, it is beneficial to form more medium and strong B acid sites, which well explains that Ni-SAPO-11 has more medium and strong B acid sites. However, in the process of *in situ* modification, Ni species uniformly enter the bulk and surface framework of SAPO-11 molecular sieves, which is limited to the increase of B acid amount. EDTA only integrates Ni species into the surface framework of SAPO-11 molecular sieves during the complexation-excessive impregnation process, and more Ni species are distributed in the surface framework of SAPO-11 molecular sieves, resulting in a significant improvement of the surface acidity of SAPO-11 molecular sieves, which is the reason why the weak B acid amount, medium and strong B acid amount of NiSAPO-11 reach their maximum. Ni-SAPO-11 modified by Ni by the complexation-excessive impregnation method initiated in this study has the largest amount of medium and strong B acid, which is beneficial to improve the catalytic performance of the catalyst.

Mechanism (1): $\left({\text{AlO}}_{4\text{/}2}\right)-\left({\text{PO}}_{4\text{/}2}\right)+\to {\left({\text{NiO}}_{4\text{/}2}\right)}^{2-}{\left({\text{PO}}_{4\text{/}2}\right)}^{+}{+\text{H}}^{+}$

Mechanism (2): $\left({\text{HOAlO}}_{3\text{/}2}\right)-{\left({\text{PO}}_{4\text{/}2}\right)}^{+}\to {\left({\text{HONiO}}_{4\text{/}2}\right)}^{2-}{\left({\text{PO}}_{4\text{/}2}\right)}^{+}{+\text{ H}}^{+}$

**TABLE 2:** Acidity properties of Ni-modified SAPO-11 determined by Py-IR.

Sample	Acidity (μmol/g)					
	Weak acid sites (200°C)			Medium and strong acid sites (350°C)		
	B	L	B + L	B	L	B + L
SAPO-11	91.87	29.32	121.19	30.62	20.97	51.59
Ni@SAPO-11	53.09	24.25	77.34	23.51	15.11	38.62
Ni-SAPO-11	69.91	35.84	105.75	48.21	18.16	66.37
NiSAPO-11	82.21	56.88	139.09	58.35	24.66	83.01

### Active Phase Characterization

**FIGURE 6:**
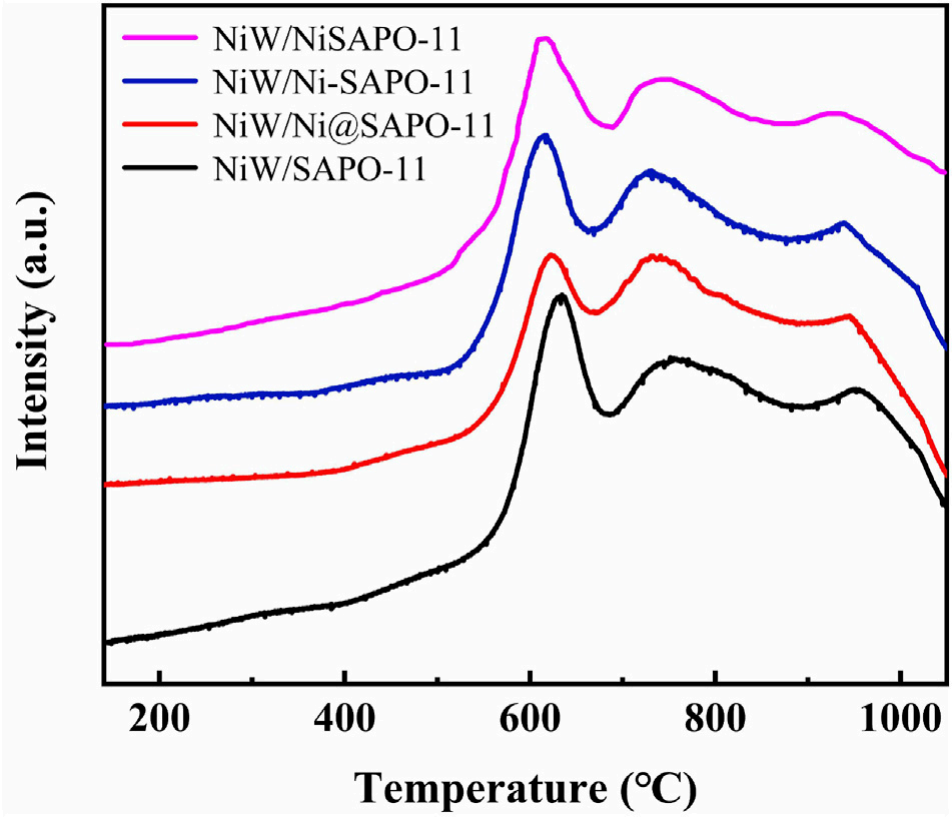
H_2_-TPR profiles of the different catalysts.

On bifunctional catalysts, the reducibility of active metals can effectively reflect the interaction between active metals and supports (Yang Z et al., 2019). As a result, H_2_-TPR characterization was used to investigate the reducibility of the active metals on the catalysts. Figure 6 depicts the H_2_-TPR profiles of all catalysts. In the range of 0–1,050°C, all samples revealed three hydrogen consumption peaks. The low-temperature reduction peak at 610–635°C is attributed to the reduction of highly dispersed octahedrally coordinated polymeric tungsten species and mostly NiO species, while the moderate-temperature reduction peak at 710–765°C is attributed to the reduction of NiWO_4_ species, and the high-temperature reduction peak at 930–955°C is attributed to the reduction of refractory tungsten species in the form of W-O-Al (Cui et al., 2013). The results revealed that the low-temperature reduction peaks, moderate-temperature reduction peaks, and high-temperature reduction peaks of the catalysts all migrated to lower temperatures after Ni alteration. The reduction temperatures of low-temperature reduction peaks decreased in the order of NiW/SAPO-11 (634°C) > NiW/Ni@SAPO-11 (624°C) > NiW/Ni-SAPO-11 (611°C) > NiW/NiSAPO-11 (608°C), and the reduction temperatures of moderate-temperature reduction peaks decreased in the order of NiW/SAPO-11 (760°C) > NiW/Ni@SAPO-11 (737°C) > NiW/Ni-SAPO-11 (726°C) > NiW/NiSAPO-11 (723°C), while the reduction temperatures of high-temperature reduction peaks decreased in the order of NiW/SAPO-11 (957°C) > NiW/Ni@SAPO-11 (944°C) > NiW/Ni-SAPO-11 (936°C) > NiW/NiSAPO-11 (922°C), indicating that the reducibility of active metal NiW on NiW/SAPO-11 catalysts can be improved by introducing Ni into SAPO-11 through the ion-exchange method or the *in situ* modification method.

**FIGURE 7:**
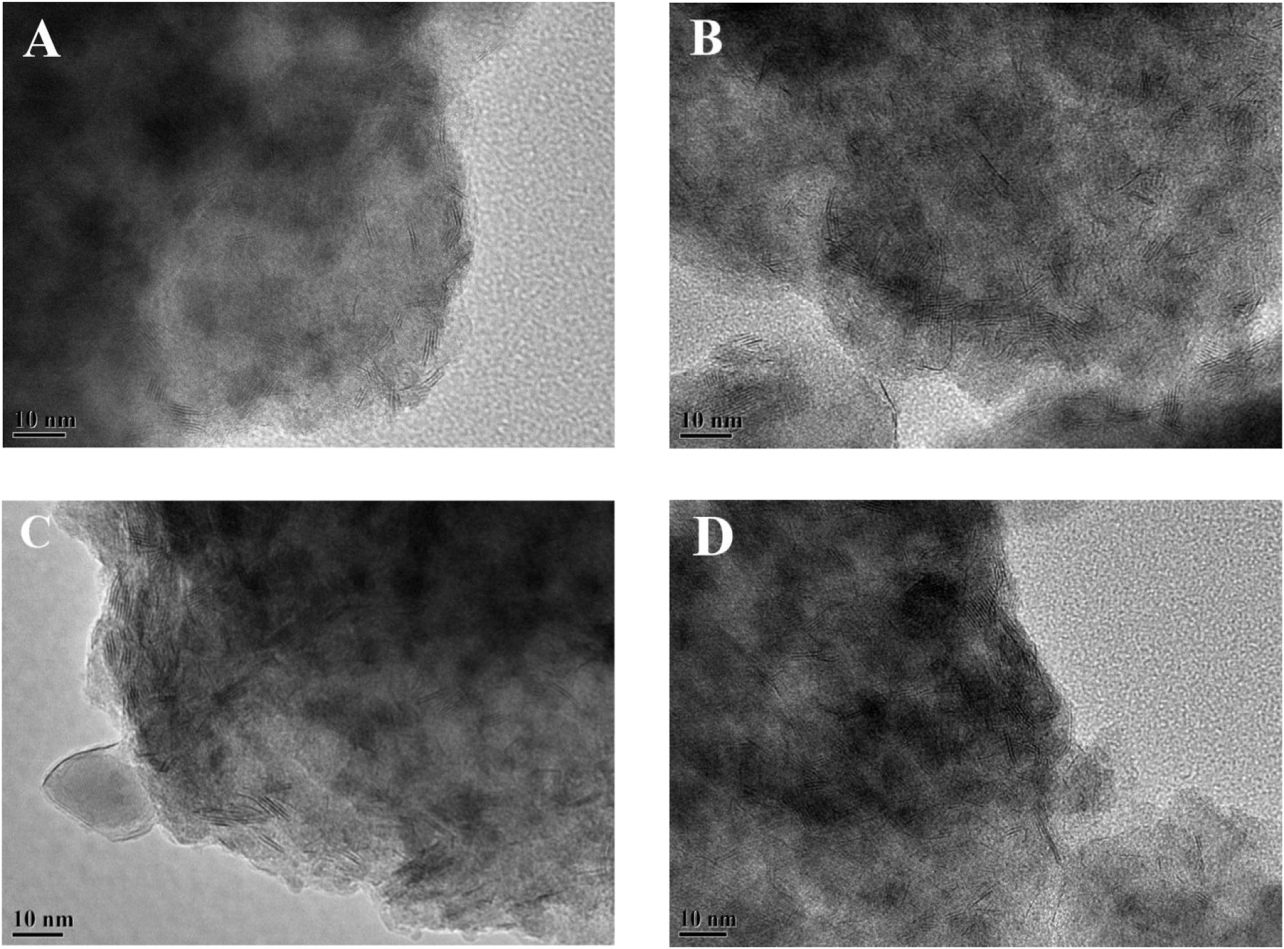
Representative HRTEM images of sulfided catalysts: **(A)** NiW/SAPO-11, **(B)** NiW/Ni@SAPO-11, **(C)** NiW/Ni-SAPO-11, and **(D)** NiW/NiSAPO-11.

The sulfided catalysts were studied by HRTEM to visualize the morphologies of the NiWS active phase on the catalysts and compare the dispersibility of sulfided metals on the various Ni-modified SAPO-11 supports. Figure 7 shows sample HRTEM images of the different supported NiW sulfided catalysts. The WS_2_ slab structure is represented by the black line-like layers in the images (Yu et al., 2012). The average length and average stacking number of WS_2_ slabs on different catalysts were calculated using statistical analysis (Eqs 1, 2) of at least 500 slabs from different regions of each sulfide catalyst, and the findings are reported in Table 3. The results showed that Ni alteration altered the morphologies of the active phase. The average length of WS_2_ slabs dropped in the sequence NiW/SAPO-11 (4.62 nm) > NiW/Ni@SAPO-11 (3.95 nm) > NiW/Ni-SAPO-11 (2.98 nm) > NiW/NiSAPO-11 (2.63 nm), whereas the average stacking number rose in the order NiW/SAPO-11 (1.33) < NiW/Ni@SAPO-11 (2.64) < NiW/Ni-SAPO-11 (3.23) < NiW.NiSAPO-11 (3.27). The length of WS_2_ slabs on NiW/SAPO-11 ranged from 1 to 7 nm, with the majority falling within the 3–6 nm range. The length of WS_2_ slabs on NiW/Ni@SAPO-11 ranged from 1 to 5 nm, with the majority falling between 2 and 5 nm. The length distribution of WS_2_ slabs on NiW/Ni-SAPO-11 was 1–4 nm, with the majority in the 2–4 nm region. The length of WS_2_ slbs of NiW/NiSAPO-11 ranges between 1–4 nm, but is mainly in the 2–3 nm range. It is often assumed that the dispersibility of the active phase is proportional to its length. The larger the dispersion degree, the shorter the active phase slabs are (Zhou et al., 2018). As a result, the degree of metal dispersion on the catalysts decreased in the following order: NiW/NiSAPO-11 > NiW/Ni-SAPO-11 > NiW/Ni@SAPO-11 > NiW/SAPO-11, which was consistent with the *f*_*W*_ values in Table 3. However, in addition to the length of WS_2_ slabs, the number of WS_2_ slabs stacked can have an impact on the properties of the active phase. The interaction between active metal and support is adequately reflected by the stacking number of WS_2_ slabs (Zhou et al., 2017). The average stacking number of WS_2_ slabs for NiW/SAPO-11 was only 1.33. There were about half of the monolayer WS_2_ slabs, which were classified as the “type I active phase” with low de/hydrogenation activity. This can be explained by the fact that when tungsten species are loaded on SAPO-11, they form strong W-O-Al bonds with the support, and the interaction is too strong. The average stacking number of WS_2_ slabs for NiW/Ni@SAPO-11 was 2.64, with around 33% of WS_2_ slabs having stacking numbers ranging from 3 to 4. This could be due to a decrease in the L acid sites of Ni@SAPO-11, resulting in a weaker interaction between active phase and support. The average stacking number of WS_2_ slabs in NiW/Ni-SAPO-11 was 3.23, with over 70% of WS_2_ slabs having stacking numbers ranging from 3 to 4. Because of their moderate dispersibility and stacking number, NiW/NiSAPO-11 slabs had an average stacking number of 3.27, and around 85% of WS_2_ slabs had 3-4 stacking layers, which are designated the “type II active phase” with good de/hydrogenation activity. This can be explained by the fact that when tungsten species are loaded on Ni-SAPO-11 and NiSAPO-11, they form W-O-Ni bonds with the support, and W-O-Ni bonds are weaker than W-O-Al bonds. Additional Ni species were coupled to the surface framework of SAPO-11 molecular sieves rather than the bulk phase during the complexation-excessive impregnation modification, resulting in the formation of more W-O-Ni species. EDTA can similarly “anchor” Ni species, allowing them to be evenly “anchored” in a specific place and hence uniformly integrated into the surface framework of the SAPO-11 molecular sieve. “Delumination and nickel supplementation” is the term for this phenomenon. Overall, among the Ni-modified SAPO-11-supported NiW catalysts, NiW/NiSAPO-11 had the best metal dispersibility and reducibility, as well as the highest hydrogenation activity.

**TABLE 3:** Average lengths, layer numbers, and *f*_*W*_ values of WS_2_ of all catalysts.

Sample	$\bar{L}$ (nm)	$\bar{N}$	*f*_*W*_
NiW/SAPO-11	4.62	1.33	0.28
NiW/Ni@SAPO-11	3.95	2.64	0.29
NiW/Ni-SAPO-11	2.98	3.23	0.31
NiW/NiSAPO-11	2.63	3.27	0.32

**FIGURE 8:**
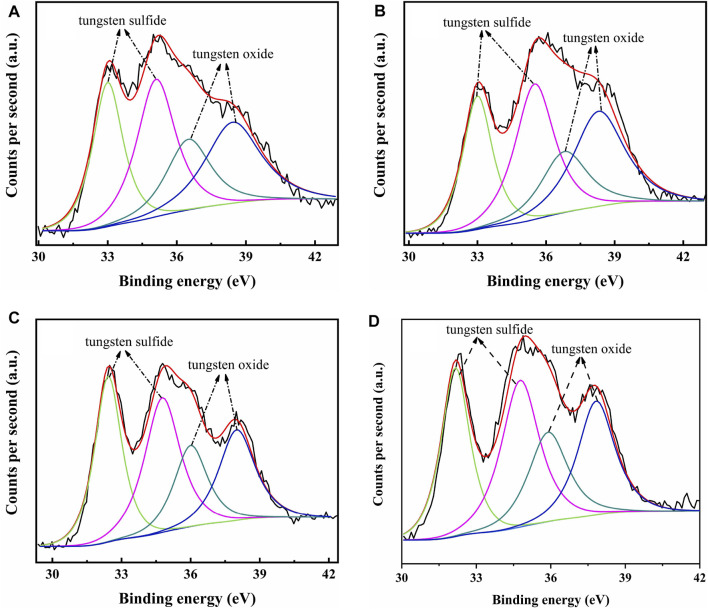
XPS W 4f spectra of sulfided catalysts: **(A)** NiW/SAPO-11, **(B)** NiW/Ni@SAPO-11, **(C)** NiW/Ni-SAPO-11, and **(D)** NiW/NiSAPO-11.

**FIGURE 9:**
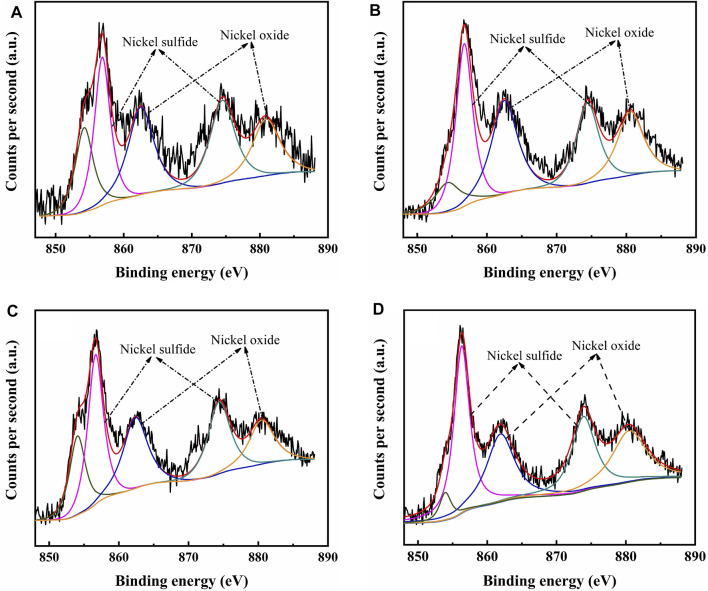
XPS Ni 2p spectra of sulfided catalysts: **(A)** NiW/SAPO-11, **(B)** NiW/Ni@SAPO-11, **(C)** NiW/Ni-SAPO-11, and **(D)** NiW/NiSAPO-11.

The catalysts were studied using XPS to determine the covalent states of nickel and tungsten surface species on the sulfided catalysts. The W 4f XPS spectra and Ni 2p XPS spectra of the sulfided catalysts are shown in Figures 8, 9, respectively, and the binding energies (BE) and Sulfidation degree of tungsten and nickel species on the sulfided catalysts are provided in Tables 4, 5, respectively. The W 4f XPS spectra were decomposed into four peaks, each of which consisted of two overlapping W^4+^ and W^6+^ peaks. The W 4f_7/2_ and W 4f_5/2_ levels of W^4+^ (WS_2_) have binding energies of around 32.70 ± 0.50 eV and 35.40 ± 0.50 eV, respectively, while the W 4f_7/2_ and W 4f_5/2_ levels of W^6+^ (WO_3_) have binding energies of about 36.50 ± 0.50 eV and 38.30 ± 0.50 eV, respectively (Cui et al., 2012). The easy metals are to sulfide, the weaker the connection between the metal and the support, and the more metal layers there are. The percentage of W^4+^ species [W^4+^/(W^4+^ + W^6+^)] has a significant impact on the catalytic performance of hydrotreating catalysts, hence the sulfidation degree of tungsten species on sulfided catalysts was estimated (Cui et al., 2013). The degree of sulfidation of tungsten species on different sulfided catalysts decreased in the order NiW/NiSAPO-11 (62.07%) > NiW/N-SAPO-11 (60.93%) > NiW/Ni@SAPO-11 (56.96%) > NiW/SAPO-11 (56.96%) > NiW/SAPO-11 (56.96%) > NiW/SAPO-11 (56.96%) > NiW/SAPO (54.29%). The decrease in interaction between active metals and support causes the increase in sulfidation degree of tungsten species on NiW/NiSAPO-11, NiW/Ni-SAPO-11, and NiW/Ni@SAPO-11 as compared to NiW/SAPO-11. To some extent, BE value can reflect the interaction between metal species and support. Table 4 shows that the BE values of tungsten species on the three catalysts, whether oxidic or sulfided, decrease in the order of NiW/SAPO-11 > NiW/Ni@SAPO-11 > NiW/Ni-SAPO-11 > NiW/NiSAPO-11. The results show that the interaction between tungsten species and support decreases in the order of NiW/SAPO-11 > NiW/Ni@SAPO-11 > NiW/Ni-SAPO-11 > NiW/NiSAPO-11, which is completely consistent with the results reflected by H_2_-TPR and HRTEM. The Ni 2p XPS spectra were separated into five peaks, including two overlapping nickel oxide and nickel sulfide peaks. The Ni 2p_3/2_ and Ni 2p_1/2_ levels of nickel oxide (NiO) have binding energies of around 862.50 ± 0.50 eV and 880.50 ± 0.50 eV, respectively, while the Ni 2p_3/2_ and Ni 2p_1/2_ levels of nickel sulfide (NiS_x_) have binding energies of about 856.50 ± 0.50 eV and 874.20 ± 0.50 eV, respectively (Yu et al., 2012). The sulfidation degree of nickel species was defined as the percentage of nickel sulfide species [NiS_x_/(NiO + NiS_x_)] (Cui et al., 2013). The sulfidation degree of nickel species on different sulfided catalysts decreased in the order of NiW/NiSAPO-11 (52.23%) > NiW/Ni-SAPO-11 (49.13%) > NiW/Ni@SAPO-11 (48.35%) > NiW/SAPO-11 (45.41%), and the explanation for this change order is the same as that of tungsten species explained above.

**TABLE 4:** Binding energy and sulfidation degree of W on different catalysts.

Sample	NiW/SAPO-11	NiW/Ni@SAPO-11	NiW/Ni-SAPO-11	NiW/NiSAPO-11
Oxidic	Binding energy (eV)			
W 4f_7_	36.70	36.50	36.10	35.82
W 4f_5_	38.43	38.30	38.00	37.78
Sulfided	Binding energy (eV)			
W 4f_7_	33.00	32.90	32.42	32.14
W 4f_5_	35.35	35.25	34.77	34.62
Sulfidation degree of W (%)	54.29	56.96	60.93	62.07

**TABLE 5:** Binding energy and sulfidation degree of Ni on different catalysts.

Sample	NiW/SAPO-11	NiW/Ni@SAPO-11	NiW/Ni-SAPO-11	NiW/NiSAPO-11
Oxidic	Binding energy (eV)			
Ni 2p_3_	862.71	862.50	862.15	861.89
Ni 2p_1_	880.90	880.57	880.26	880.11
Sulfided	Binding energy (eV)			
Ni 2p_3_	856.84	856.74	856.65	856.35
Ni 2p_1_	874.51	874.42	874.31	873.88
Sulfidation degree of Ni (%)	45.41	48.35	49.13	52.23

### Catalytic Performance

**FIGURE 10:**
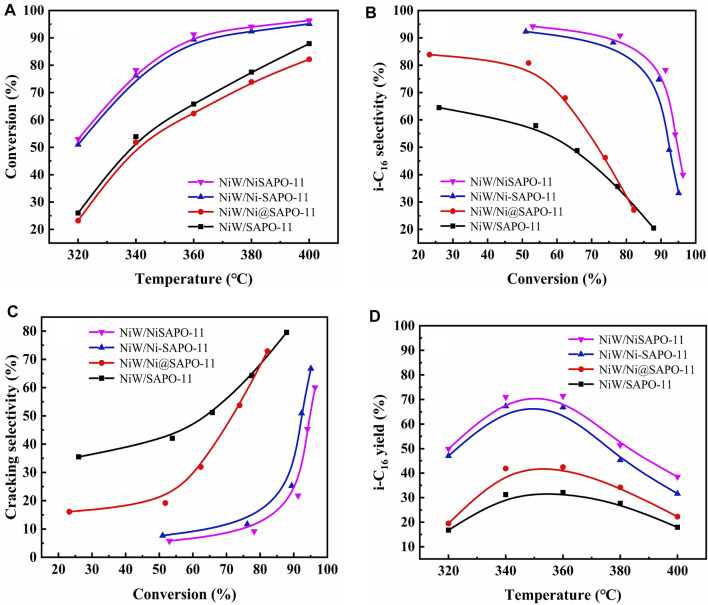
Catalytic performance of the different catalysts: **(A)** n-C_16_ conversion, **(B)** i-C_16_ selectivity, **(C)** cracking selectivity, and **(D)** i-C_16_ yield.

The catalyst was sulfurized ahead of time, and the reaction pressure was set to 2.0 MPa, LHSV was set at 1.5 h^−1^, and H_2_/oil was 600 (v/v) in a typical reaction. In the reaction temperature range of 300–400°C, the catalytic performance of the catalyst was studied. Figure 10A depicts n-C_16_ conversion on several catalysts at various reaction temperatures. The conversion on all catalysts increased with the increase in reaction temperature, as shown in Figure 10A. The catalytic activity of NiW/NiSAPO-11 was much higher than that of other catalysts. The catalytic activity of all the catalysts increased in the order of NiW/Ni@SAPO-11 < NiW/SAPO-11 < NiW/Ni-SAPO-11 < NiW/NiSAPO-11. The selectivity of all catalysts to i-hexadecane (i-C_16_) reduced as reaction temperature climbed, showing that the rate of cracking side reactions increased as reaction temperature increased Figure 10C. The selectivity of NiW/NiSAPO-11 to i-C_16_ was clearly higher than that of NiW/SAPO-11, NiW/Ni@SAPO-11, and NiW/Ni-SAPO-11 within the reaction temperature range of catalytic performance evaluation (Figure 10B). This can be explained by the fact that NiSAPO-11 has the most medium and strong B acid sites, which are universally recognized as active sites for the isomerization of olefin intermediate skeletons (Yu et al., 2021). Furthermore, on NiW/Ni-SAPO-11, the interaction between active metals and support is weak, and the dispersibility and stacking number of the so-called NiWS active phase are moderate, resulting in good hydrogenation activity for isomeric olefin intermediates that diffuse from the acid to the metal sites. The interaction between the active metals and the support in NiW/SAPO-11 is strong, making it difficult to diminish the active metals and resulting in lower i-C_16_ selectivity than NiW/Ni-SAPO-11. NiW/Ni@SAPO-11 has the fewest medium and strong B acid sites, but its active phase dispersion degree and stacking number are higher than those of NiW/SAPO-11, resulting in lesser n-C_16_ conversion and higher isomer selectivity than NiW/SAPO-11. Figure 10D shows the yield of i-C_16_ for various catalysts, and the yield of i-C_16_ for NiW/NiSAPO-11 is clearly higher than the other three catalysts. The maximum yield of i-C_16_ of different catalysts increased in the order of NiW/SAPO-11 (32.10%) > NiW/Ni@SAPO-11 (42.41%) > NiW/Ni-SAPO-11 (66.82%) > NiW/NiSAPO-11 (71.40%). These results indicate that the complexation-excessive impregnation-modified SAPO-11-supported NiWS catalyst has a better catalytic performance than that of ion-exchange modified SAPO-11 support NiWS catalyst, unmodified SAPO-11-supported NiWS catalyst, and *in situ* Ni-modified SAPO-11 support NiWS catalyst for n-hexadecane hydroisomerization.

The isomer product distributions, TOF values and k_iso_ values of different catalysts at 340°C are presented in Table 6. The reaction rate constants of different catalysts increased in the order of NiW/Ni@SAPO-11 (5.80 × 10^–3^ mol g^−1^ h^−1^) < NiW/SAPO-11 (7.26 × 10^–3^ mol g^−1^ h^−1^) < NiW/Ni-SAPO-11 (1.16 × 10^–2^ mol g^−1^ h^−1^) < NiW/NiSAPO-11 (1.21 × 10^–2^ mol g^−1^ h^−1^), which is due to the difference between the amount of medium and strong B acid in the three supports. While the TOF values of the three catalysts decreased in the order of NiW/NiSAPO-11 (31.22 h^−1^) > NiW/Ni-SAPO-11 (30.41 h^−1^) > NiW/Ni@SAPO-11 (26.72 h^−1^) > NiW/SAPO-11 (26.29 h^−1^), and the calculated TOF values well verified the catalytic activity of the catalysts. This result can be explained by the fact that the weakening of the interaction between the active phase and the support promoted more active phases to form “type II active phase”, which increased the desorption rate of carbocation on the active phase. The isomer products of different catalysts were mainly mono-branched isomer products, and monomethyl branched isomer products were the main mono-branched isomer products.

**TABLE 6:** Isomer distributions, TOF values, and k_iso_ values of n-C_16_ hydroisomerization over the different catalysts at 340 °C.

Products	Selectivity (%)			
	NiW/SAPO-11	NiW/Ni@SAPO-11	NiW/Ni-SAPO-11	NiW/NiSAPO-11
Selectivity of C_16_ (%)	57.92	80.81	88.30	90.81
TOF (h^−1^)	26.29	26.72	30.41	31.22
k_iso_ (mol g^−1^ h^−1^)	7.26 × 10^–3^	5.80 × 10^–3^	1.16 × 10^–2^	1.21 × 10^–2^
2-MC_15_	13.57	16.99	17.87	19.77
3-MC_15_	13.65	19.07	20.43	18.25
4-MC_15_	7.17	12.74	13.27	16.31
5-MC_15_	7.18	8.81	10.01	8.78
6-MC_15_	4.54	5.41	5.95	6.17
2,4-DMC_14_	3.10	4.18	5.72	5.69
2,5-DMC_14_	2.92	4.23	4.91	4.42
2,6-DMC_14_	1.98	3.31	3.35	3.87
3,6-DMC_14_	1.13	1.88	2.37	3.03
3,7-DMC_14_	0.77	1.21	1.38	1.77
3-EC_14_	0.43	0.73	0.76	1.13
5-EC_14_	0.38	0.78	0.83	0.97
6-EC_14_	0.32	0.55	0.48	0.55
5,8-DEC_12_	0.28	0.44	0.25	0.45
Others	0.50	0.48	0.72	0.65

## Conclusion

The ion-exchange method, *in situ* synthesis method, and complexation-excessive impregnation modification method successfully prepared Ni-modified SAPO-11 molecular sieves, and the corresponding NiW-supported catalysts were successfully prepared by the incipient-wetness impregnation method, and then used for hydroisomerization of n-hexadecane. The effects of several Ni-modification procedures on the properties of SAPO-11, particularly the active phase properties on the related catalysts, were studied. The results showed that the crystal structure of Ni@SAPO-11 prepared by the ion-exchange method collapsed partially, and nickel species occupied a portion of the pore volume and covered a portion of the acid sites, resulting in a drop in specific surface area, pore volume, and acid sites amount. Ni-SAPO-11 produced *in situ* had a larger specific surface area, pore volume, and medium and strong Brønsted acid sites. However, during dealumination, EDTA had the effect of dredging channels and reaming holes, resulting in a larger BET specific surface area and pore volume of NiSAPO-11. Furthermore, EDTA made it simple to incorporate Ni species into the surface framework of the SAPO-11 molecular sieve rather than the bulk phase, resulting in NiSAPO-11 with better acidity. The support of NiW/NiSAPO-11 had the most Brønsted acid sites, the weakest interaction between active metals and support, and the highest dispersibility and stacking number of active phase, all of which were helpful to n-hexadecane hydroisomerization. NiW/NiSAPO-11 had much greater n-C_16_ conversion, i-C_16_ selectivity, and i-C_16_ yield than the other catalysts. It is expected to provide theoretical guidance for the design of high-activity non-noble metal catalysts for the hydroisomerization of alkanes.

## Data Availability

The original contributions presented in the study are included in the article/Supplementary Material, further inquiries can be directed to the corresponding author.
